# Thrombin generation indices and Wells score predict pulmonary embolism in patients with acute exacerbation of chronic obstructive pulmonary disease

**DOI:** 10.1016/j.clinsp.2025.100582

**Published:** 2025-02-28

**Authors:** Linjie Luo, Dan Zheng, Li Da, Jian Cheng, Yirui Cao, Na Wang

**Affiliations:** Department of Respiratory and Critical Care medicine, The Affiliated Hospital, Southwest Medical University, Chengdu City, Sichuan Province, PR China

**Keywords:** Thrombin Generation, Wells Score, AECOPD, PE

## Abstract

**Objective:**

This study investigated the predictive value of thrombin generation indices and Wells score in the development of Pulmonary Embolism (PE) in patients with Acute Exacerbation of Chronic Obstructive Pulmonary Disease (AECOPD).

**Methods:**

160 patients who were admitted for AECOPD and underwent CT Pulmonary Arteriography (CTPA) were collected. Among them, a total of 62 cases were diagnosed with PE as the AECOPD with PE group, and the other 98 cases were not diagnosed with PE as the AECOPD group. The general data, past history and combined basic diseases, laboratory tests and other related clinical data of the two groups were compared, and the data collected were statistically analyzed to explore the diagnostic indexes that can predict PE in AECOPD.

**Results:**

History of venous thromboembolism, d-Dimer as well as Endogenous Thrombin Potential (ETP), Activated Partial Thromboplastin Time (APTT) coagulation indices, and Wells score was significantly higher in the AECOPD with PE group than in the AECOPD group, and Time to Peak (ttpeak), Albumin (ALB) and total protein were lower than those in the AECOPD group. The Wells score had a positive correlation with ETP and APTT and a negative correlation with ttpeak, which were all independent risk factors for PE in AECOPD. The Wells score had the best efficacy in predicting the occurrence of PE in patients with AECOPD with a cutoff value of 4.62.

**Conclusion:**

Significant correlations were found between the thrombin generation indices (ETP, APTT and ttpeak) and the Wells score, which can also be used for early diagnosis of PE in patients with AECOPD.

## Highlights

• History of venous thromboembolism, d-Dimer as well as ETP, APTT coagulation indices, and Wells score were significantly higher in the AECOPD with PE group, and ttpeak, ALB and TP were lower.
• Wells score had a positive correlation with ETP and APTT and a negative correlation with ttpeak, which were all independent risk factors for PE in AECOPD.
• Wells score, ETP, APTT and ttpeak had the efficacy of predicting PE by ROC curve analysis.

## Introduction

Exacerbations of Chronic Obstructive Pulmonary Disease (COPD) are due to changes in pathophysiology, such as increased airway and systemic inflammation, leading to worsening respiratory symptoms.^1^ Acute Exacerbation of COPD (AECOPD) is a common reason for emergency room visits and is defined as an acute worsening of symptoms beyond daily variability that requires additional therapy.^2^ Most COPD-related deaths occur during acute exacerbations. Pulmonary Embolism (PE) is one of the common complications of AECOPD. The risk of PE and other Venous Thromboembolism (VTE) events in patients with COPD is approximately twice as high as that of patients without COPD.^3^ Because PE may present similarly to AECOPD, it is often difficult for patients with COPD to differentiate between the two diseases.^4^ Currently, the diagnosis of PE based on clinical suspicion alone is not accurate.

Circulatory and coagulation dysfunction frequently occurs when patients with AECOPD develop respiratory failure. Abnormal coagulation and thrombosis may accelerate AECOPD.^5^ In addition, elevated levels of coagulation factors and endothelial damage to the pulmonary vasculature may increase pulmonary vascular resistance, and obstruction and vasculopathy may occur,^6^^,^^7^ leading to an increased risk of chronic thrombo-embolic pulmonary disease or chronic thromboembolic pulmonary hypertension.^8^, ^9^, ^10^ Studies have shown that blood hypercoagulability, blood stasis, and vascular endothelial damage are the three major causative factors for thrombosis.^11^^,^^12^ The blood in AECOPD patients is hypercoagulable, thus causing thrombosis.^13^, ^14^, ^15^ Routine coagulation tests detect the presence of abnormalities in endogenous and exogenous coagulation pathways.^16^ The Calibrated Automated Thrombogram (CAT) is a process that activates the complete generation of thrombin by enrichment of tissue factors, and captures the full spectrum of thrombin action.^17^^,^^18^ CAT is a powerful tool for the detection of Endogenous Thrombin Potential (ETP), Thrombin Peak Height (TPH), and time to peak (ttpeak). All of these indicators have been previously used to detect blood coagulation status and function in the early stages of sepsis, recurrent VTE, and other diseases.^19^, ^20^, ^21^, ^22^ However, most of the current studies have focused on exploring the correlation between blood hypercoagulability and AECOPD, and there is a lack of research on thrombin generation and PE in AECOPD patients.

The Wells score, as the earliest proposed PE scoring scale, is more mature than other scoring scales and is widely used. This method comprehensively assesses the likelihood of acute PE by combining patients' symptoms, signs, and PE risk factors.^23^ A higher Wells score indicates a higher probability of VTE, but the assessment is limited due to subjectivity. The Wells score can be assessed by a two-group method or a three-group method, with the two-group method being simpler and more widely used. The apparent advantages of the three-group assessment have not been demonstrated. Despite the superiority of the Wells score over other scoring systems, a small amount of Deep Vein Thrombosis (DVT) is still present in the low probability group.^24^

The endothelial damage and hypercoagulability of the pathophysiologic process of AECOPD can lead to PE, and the pathogenesis of PE is relatively insidious, with a lack of specificity of symptoms and a low detection rate. Therefore, this study aims to investigate the value and diagnostic efficacy of thrombin generation and Wells score in the early diagnosis of PE.

## Materials and methods

### *Study patients*

According to the inclusion and exclusion criteria, 160 patients with AECOPD diagnosed in Wenjiang District People's Hospital of Chengdu from April 2021 to April 2023 were selected. They were categorized into 62 cases in the AECOPD with the PE group and 98 cases in the AECOPD group. The age of the patients in the PE group ranged from 61‒75-years-old, with a mean age of (68.10±7.10) years. The age of the patients in the AECOPD group ranged from 62‒74-years-old, with a mean age of (68.11±7.08) years. The study was approved by the Wenjiang District People's Hospital of Chengdu Ethics Committee and all study subjects voluntarily signed an informed consent form. All methods in the study were carried out in accordance with the STARD guidelines.

### *Research design*

This study is a case-control study. The general data, laboratory index test data, and comorbidities of the two groups were collected. The specific clinical characteristics and manifestations of PE in AECOPD patients were summarized, and the risk factors were analyzed. The Receiver Operating Characteristic (ROC) curve was used to analyze the indicators with clinical diagnosis, differential diagnosis, or predictive value.

### *Inclusion criteria*

(1) All patients met the diagnostic criteria of the Global Initiative for Chronic Obstructive Lung Disease (GOLD 2023). (2) PE patients met the relevant criteria of the Guideline for the Diagnosis and Treatment and Prevention of Pulmonary Thromboembolism.

### *Exclusion criteria*

(1) Patients with no previous definitive diagnosis of COPD and no acute exacerbation on this admission. (2) Patients with malignant tumors. (3) Patients with contraindications to undergoing Computed Tomography Pulmonary Angiography (CTPA). (4) Patients with severe abnormalities of liver, kidney, or heart function. (5) Patients with previous stroke or coagulation disorders. (6) Patients with communicable diseases. (7) Patients with immunologic diseases.

### *AECOPD diagnostic criteria*

The diagnostic criteria for AECOPD refer to the Global Initiative for Chronic Obstructive Lung Disease (GOLD 2023) developed in 2023.^25^ The main manifestations were aggravation of subjective symptoms, sudden changes of respiratory symptoms (dyspnea, cough aggravation, increased sputum volume, and yellow purulent sputum), and requirements for additional treatment.

### *PE diagnostic criteria*

PE diagnostic criteria refer to the 2019 Guidelines of the European Society of Cardiology on the diagnosis and management of acute pulmonary embolism.^26^ Clinical presentation is consistent with pulmonary thromboembolism; CTPA examination shows complete or incomplete filling defects within the pulmonary arteries and distal vessels are not visualized. The lung field was characterized by wedge-shaped, striated hyperdense shadows or discoidal lung atelectasis, with central pulmonary artery dilatation and reduced or absent distal vascular branching. Echocardiography shows thrombus in the right cardiac system including the right atrium, right ventricle, and pulmonary artery.

### Observation indicators

General information about the patients was collected, including gender, age, Body Mass Index (BMI), and co-morbidities, such as DVT, varicose veins of the lower extremities, hypertension, coronary artery disease, and diabetes mellitus.

White Blood Cell (WBC), Red Blood Cell (RBC), Hemoglobin (HGB), Platelet Crit (PCT), percentage of Neutrophils (NEUT), serum Albumin (ALB), and Total Protein (TP) were measured on a fully automated blood cell analyzer (BC-6800Plus; Mindyray, Shenzhen, China). Triacylglycerol (TG), Low-Density Lipoprotein Cholesterol (LDL-C), High-Density Lipoprotein Cholesterol (HDL-C), and total Cholesterol (TC) were analyzed using an automatic biochemistry analyzer (AU680, Beckman Coulter; Shanghai, China).

Fasting venous blood (3 mL) was taken from patients within 24 h of admission to the hospital, and the thrombin generation indexes were detected by CAT method on a fluorescence reader (model Fluoroskan Ascent FL; Thermo Scientific, MA, USA), and the TG curve was generated by CAT software. (1) TPH: the value when the TG curve reaches the highest; (2) ttpeak: the time from the beginning of the reaction to the peak of the TG curve; (3) ETP: the calculus area under the TG curve, reflecting the overall thrombin generation.

Fasting peripheral fasting venous blood (4 mL) was centrifuged at 2000 r/min for 30 min, and the plasma specimen was placed at −80 °C. d-Dimer, Activated Partial Thromboplastin Time (APTT), fibrinogen was measured using a fully automated hemagglutination analyzer (MC-4000; MDC Medicine Devices, Beijing, China).

### *Wells score*

Seven items were evaluated, including symptoms and signs of DVT, history of DVT or PE, heart rate >100 beats/min, history of major surgery or trauma in the last 4-weeks, hemoptysis, and malignancy, with higher scores indicating a higher risk of venous thrombosis. The patients were grouped according to the Wells score, < 2 was categorized as 12 cases in the Low Risk (LR) group, 2 to 6 was categorized as 144 cases in the Moderate Risk (MR) group, and > 6 was categorized as 4 cases in the High Risk (HR) group.

### *Statistical analysis*

The experimental data were statistically analyzed using SPSS 26.0. Measurement information obeying normal distribution was expressed as mean ± standard deviation, two-group comparisons were made using *t*-test, and multiple comparisons were made using Tukey's multiple comparison test. Information that did not obey normal distribution was expressed as M (Q25∼Q75), and comparisons between groups were made using Wilcoxon signed rank sum test. Correlation analysis obeying normal distribution was performed using Pearson's correlation and corrected for p-value using Bonferroni. The significance level was α = 0.05. Qualitative information was expressed as number of cases and percentage (%) using the Chi-Square test. Multifactorial logistic regression was used to analyze a variable as an independent risk factor for PE in AECOPD patients. The value of thrombin generation indices and Wells score in predicting PE in patients with AECOPD was assessed using the ROC curve and Area Under the Curve (AUC); *p* < 0.05 was considered a statistically significant difference.

## Results

### *Clinical general information*

General information is presented in Table 1. The prevalence of DVT and d-Dimer concentration in the AECOPD with PE group were higher than that in the AECOPD group, while ALB and TP were lower (all *p* < 0.05). The comparison of lipid metabolism indexes (TC, TG, LDL-C, HDL-C) as well as blood routine (WBC, RBC, HGB, PCT, NEUT) between the two groups of patients showed no statistically significant differences (all *p* > 0.05).

**Table 1:** Comparison of clinical general data between AECOPD with PE group and AECOPD group.

Characteristics	AECOPD with PE (*n* = 62)	AECOPD (*n* = 98)	p-value
Male	33 (53 %)	51 (52 %)	0.854
Age (year)	68.10 ± 7.10	68.12 ± 6.08	0.112
BMI (kg/m^2^)	21.82 ± 1.17	22.14 ± 1.23	0.208
Comorbidity			
Deep vein thrombosis	19 (30 %)	5 (5 %)	0.00
Varicose vein of lower limb	5 (8 %)	6 (6 %)	0.425
Hypertension	31(50 %)	48 (49 %)	0.098
Diabetes	9 (13 %)	11 (11 %)	0.123
Coronary heart disease	17 (27 %)	33 (32 %)	0.64
Smoking history	18 (28 %)	21 (21 %)	0.522
d-Dimer(μg/mL)	3.25 ± 0.53	1.02 ± 0.18	<0.001
RBC (*10^12^/L)	4.29 (3.92, 4.78)	4.57 (4.15, 4.95)	0.251
PCT (%)	0.23 ± 0.058	0.22 ± 0.052	0.259
WBC (*10^9^/L)	7.31 ± 3.65	7.12 ± 3.24	0.731
HGB (g/L)	127.97 ± 16.25	130.55 ± 18.54	0.37
ALB (g/L)	34.70 (32.38, 38.23)	36.50 (33.60, 39.08)	0.025
TP (g/L)	60.50 (58.68, 63.28)	63.00 (59.63, 66.70)	0.031
NEUT	66.58 ± 11.46	65.81 ± 12.15	0.69
FIB (mmoL/L)	3.30 (2.47, 4.21)	3.37 (2.6, 4.51)	0.483
TC (mmoL/L)	5.18 ± 1.14	5.05 ± 1.26	0.511
TG (mmoL/L)	1.64 ± 0.49	1.50 ± 0.55	0.104
LDL-C (mmoL/L)	2.34 ± 0.62	2.33 ± 0.71	0.928
HDL-C (mmoL/L)	1.26 ± 0.41	1.22 ± 0.32	0.492

Measurement data were expressed as mean ± standard deviation or n (%). When the data does not conform to the normal distribution, it is expressed as M (Q25∼Q75). Continuous variables conforming to the normal test were tested by Student *t*-test, and categorical variables were tested by Chi-Square test (*p* < 0.05).

AECOPD, Acute Exacerbation of Chronic Obstructive Pulmonary Disease; PE, Pulmonary Embolism; BMI, Body Mass Index; RBC, Red Blood Cell; PCT, Platele Crit; WBC, White Blood Cell; HGB, Haemoglobin; ALB, Serum Albumin; TP, Total Protein; NEUT, Percentage of Neutrophils; FIB, Fibrinogen; TC, Total Cholesterol; TG, Triglyceride; LDL-C, Low-Density Lipoprotein Cholesterol; HDL-C, High-Density Lipoprotein Cholesterol.

### *Serum thrombin generation parameters and wells score in aecopd with pe and aecopd groups*

**Fig. 1:**
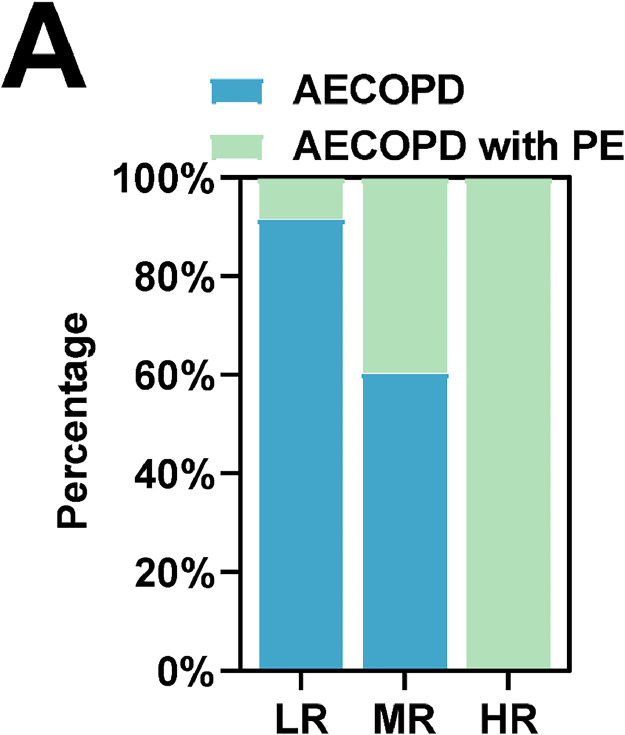
**Accuracy of Wells score for PE.** AECOPD, Acute Exacerbation of Chronic Obstructive Pulmonary Disease; PE, Pulmonary Embolism; LR, Low Risk; MR, Moderate Risk; HR, High Risk.

In the AECOPD with PE group, ETP, APTT, and Wells scores were higher than in the AECOPD group, while ttpeak was lower (*p* < 0.05) (Table 2). Wells score was assessed in 160 patients with AECOPD, which was divided into three main categories, LR, MR, and HR. Of the 12 patients assessed in the LR group, 8.33 % were in the AECOPD with PE group. Of the 144 patients assessed as MR, patients in the AECOPD with PE group accounted for 39.58 %. Patients who were considered HR were diagnosed with PE (Fig. 1A).

**Table 2:** Comparison of serum thrombin generation parameters between two groups of patients.

Characteristics	AECOPD with PE (*n* = 62)	AECOPD (*n* = 98)	p-value
ETP [nmoL/(L·min)]	273.2 ± 56.32	236.7 ± 57.5	<0.001
ttpeak (min)	15.49 ± 3.16	18.51 ± 4.12	<0.001
TPH (nmoL/L)	72.51 ± 20.48	76.05 ± 25.41	0.357
APTT (s)	31.65 ± 4.14	30.35 ± 3.29	0.029
Wells score	4.90 ± 1.02	4.26 ± 1.06	<0.001

Data are expressed as mean ± standard deviation. Student *t*-test was used for statistical analysis (*p* < 0.05).

AECOPD, Acute Exacerbation of Chronic Obstructive Pulmonary Disease; PE, Pulmonary Embolism; ETP, Endogenous Thrombin Potential; TPH, Thrombin Peak Height; ttpeak, Time to peak; APTT, Activated Partial Thromboplastin Time.

### *Multifactorial logistic regression analysis of factors influencing the occurrence of PE*

**Fig. 2:**
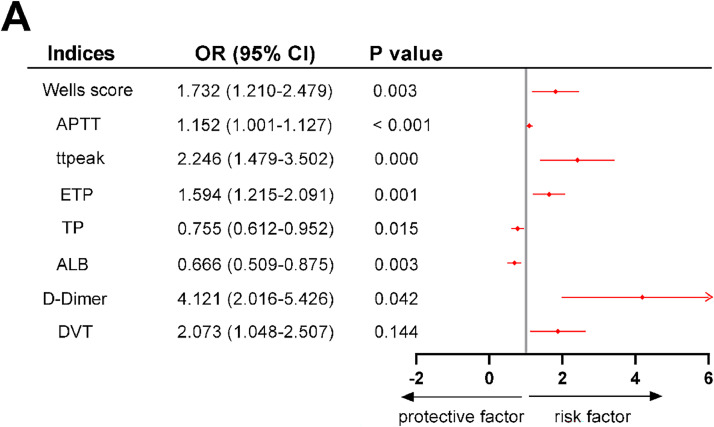
A forest plot based on multifactorial logistic regression analysis to analyze the independent factors influencing the occurrence of PE (*p<0.05). APTT, Activated Partial Thromboplastin Time; ttpeak, Time to peak; ETP, Endogenous Thrombin Potential; TP, Total Protein; ALB, Serum Albumin; DVT, Deep Vein Thrombosis; OR, Odds Radio; CI, Confidence Interval.

With DVT, d-Dimer, ALB, TP, ETP, APTT, ttpeak, and Wells score as independent variables and groups as dependent variables (AECOPD with PE group = 1, AECOPD group = 0), a multifactorial logistic regression model was set up for analysis. As Fig. 2A showed, ETP (OR [95 % CI] 1.594 [1.215‒2.091], *p* < 0.05), APTT (OR [95 % CI] 1.152 [1.001‒1.127], *p* < 0.05), ttpeak (OR [95 % CI] 2.246 [1.479–3.502], *p* < 0.05), and Wells score (OR [95 % CI] 1.732 [1.210‒2.479], *p* < 0.05) were independent risk factors for PE in patients with AECOPD. In addition, ALB (OR ([95 % CI] 0.666 [0.509‒0.879], *p* < 0.05) and TP (OR [95 % CI] 0.755 [0.612‒0.952], *p* < 0.05) were independent protective factors for developing PE in AECOPD patients, and d-Dimer (OR [95 % CI] 4.121 [2.016‒5.426], *p* < 0.05) was an independent risk factor for developing PE in AECOPD patients.

### *Correlation analysis of ETP, aptt and ttpeak with wells score*

**Fig. 3:**
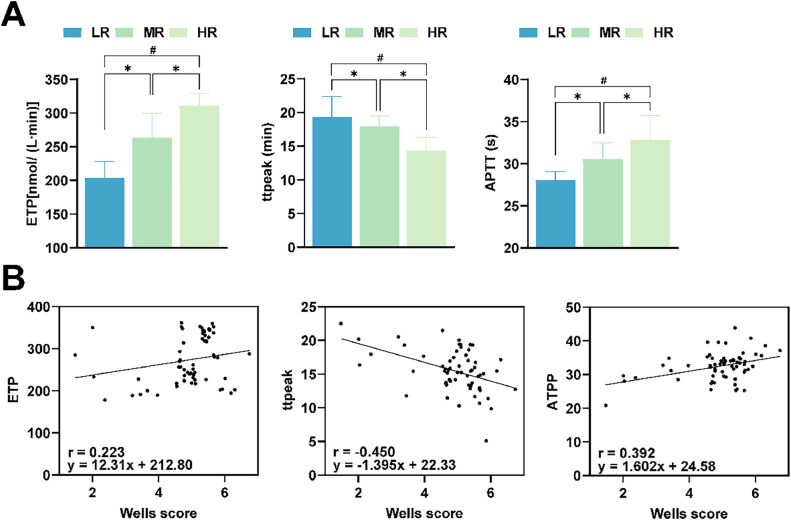
**Correlation analysis between Wells score and ETP, APTT, and ttpeak levels.** (A) Comparison of ETP, APTT and ttpeak between LR, MR, and HR groups; (B) Pearson analysis of the correlation between Wells score and ETP, APTT, and ttpeak. *p < 0.05, indicates significant difference between two groups; ^#^*p* < 0.05, indicates significant difference between three groups. LR, Low Risk; MR, Moderate Risk; HR, High Risk; ETP, Endogenous Thrombin Potential; ttpeak, Time to peak; APTT, Activated Partial Thromboplastin Time.

ETP, APTT, and ttpeak in the LR group were lower than those in the MR and HR groups, and APTT and ttpeak in the MR group were lower than those in the HR group (*p* < 0.05, Fig. 3A). Pearson results showed that APTT (*r* = 0.392, *p* < 0.05) and ETP (*r* = 0.223, *p* < 0.05) were positively correlated with Wells score in AECOPD patients. And ttpeak (*r* = −0.450, *p* < 0.05) was negatively correlated with Wells score (Fig. 3B).

### *Predictive efficacy of ETP, APTT, ttpeak, with wells score for the occurrence of pe in patients*

**Fig. 4:**
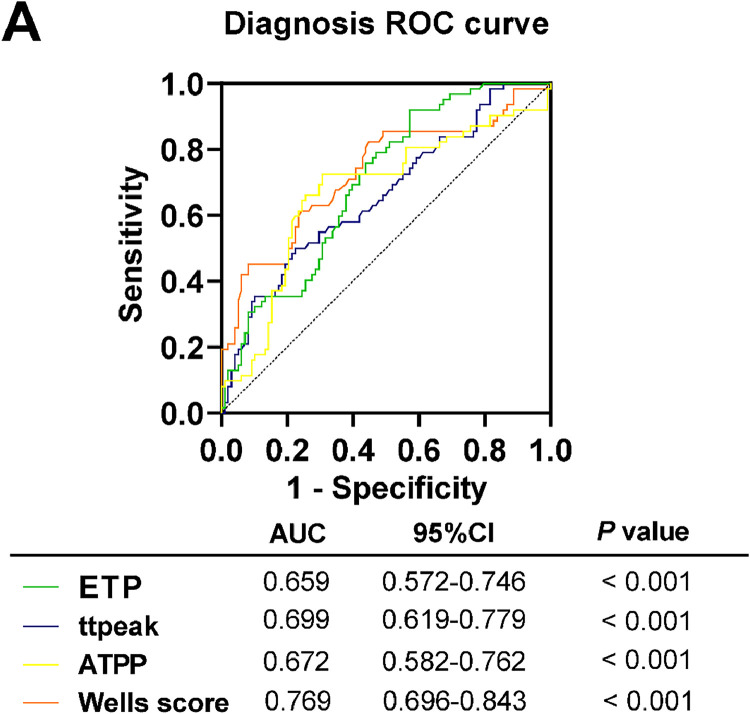
ROC curve analysis of the predictive value of ETP, TPH, APTT, and ttpeak and Wells score for PE in patients (*p<0.05). ETP, Endogenous Thrombin Potential; ttpeak, Time to peak; APTT, Activated Partial Thromboplastin Time; ROC, Receiver Operating Characteristic; AUC, Area Under the Curve; CI, Confidence Interval.

ROC curves were plotted with the AECOPD with PE group as positive samples and the AECOPD group as negative samples, and the occurrence of PE in patients as a status variable (1 = Occurrence, 0 = Non-occurrence). The ROC curves showed a higher predictive efficacy for Wells score. The AUC of Wells score was 0.728 (*p* < 0.05), and when the cut-off value was taken as Wells score > 4.62, the sensitivity of predicting the occurrence of PE was 80.65 % and the specificity was 56.12 %. The predictive efficacy of ETP, ttpeak and ATPP was relatively weak, with AUC of 0.659, 0.6988 and 0.672, the sensitivity of 85.48 %, 91.94 % and 72.58 %, and specificity of 50.00 %, 42.86 % and 69.39 %, respectively (*p* < 0.05) (Table 3, Fig. 4A).

**Table 3:** The predictive value of thrombin generation and Wells score for PE in AECOPD patients.

Indices	Cut-off	Sensitivity (%)	Specificity (%)	p-value
ETP	261.8	85.48	50.00	<0.001
ttpeak	16.76	62.90	77.55	<0.001
APTT	30.72	72.85	69.39	<0.001
Wells score	4.62	80.65	56.21	<0.001

Receiver operating characteristic curve was used to evaluate the value of thrombin genration and Wells score in predicting PE in AECOPD patients (*p* < 0.05).

AECOPD, Acute Exacerbation of Chronic Obstructive Pulmonary Disease; PE, Pulmonary Embolism; ETP, Endogenous Thrombin Potential; ttpeak, Time to peak; APTT, Activated Partial Thromboplastin Time.

## Discussion

Coagulation disorders are common in COPD patients. APTT and d-Dimer were commonly used indicators for clinical testing of coagulation function, which can directly reflect the levels of endogenous and exogenous coagulation factors. Wells score is a widely used PE scoring scale. In this study, ETP, TPH, and APTT of patients in the AECOPD with PE group were lower than those in the AECOPD group, while the ttpeak and Wells scores were higher than those in the AECOPD group. That is, thrombin generation in PE patients was less than that in AECOPD patients, and the time to reach the maximum peak thrombin was longer. This study showed that the occurrence of PE in AECOPD patients was significantly correlated with abnormal expression of coagulation indices ETP, APTT, ttpeak and Wells score with predictive efficacy.

The presence of abnormally elevated lipid levels, hemoconcentration, hypoproteinemia, fibrinolytic dysfunction, coagulation dysfunction, and endothelial dysfunction has been linked to hypercoagulable states.^27^ As one of the molecular markers of hypercoagulable state and high fibrinolysis *in vivo*, d-Dimer is a specific degradation product of cross-linked fibrin, which can better reflect the production of thrombin and the activity of fibrinolysis. However, a number of clinical conditions increase d-Dimer concentrations, such as infections, cancer, and surgery.^28^ The hypercoagulable state that occurs in AECOPD may also increase d-Dimer levels.^29^ Therefore, it is difficult to differentiate between PE and AECOPD by d-Dimer levels alone. To assess the coagulation phenotype of individual patients, the authors used CAT which can quantify plasma prothrombin kinetics. CAT is a rapid and sensitive method to quantify plasma thrombin production in real-time in response to tissue factors and procoagulant phospholipids. More importantly, total thrombin generation has been shown to be an independent predictor of VTE.^30^ Theoretically, a “hypercoagulable blood state” is reflected by a higher peak and shorter ttPeak, and the results of the present study are consistent with previous studies in that patients with PE had greater levels of ETP and ATPP and shorter ttPeak. Also consistent with other lung disease states, such as asthma, prothrombotic effects are associated with coagulation abnormalities. Elevated levels of coagulation-related factors, such as ETP, in patients with asthma are positively correlated with the severity of the disease.^31^ This evidence suggests that thrombin generation indices markers play an important role in PE and other thromboembolism-related diseases, and may provide valuable information for predicting disease prognosis, identifying coagulation abnormalities, and potentially developing targeted therapeutic strategies to improve patient prognosis.

Both thrombin generation indices and Wells score were independent risk factors for PE in AECOPD patients and had diagnostic efficacy, suggesting their effectiveness in the initial diagnostic screening for PE. Wells score, as a classic score for PE, has been evaluated by many scholars in the past for its ability to predict PE. In a retrospective study, the Wells score is more predictive of PE in patients with DVT than the Geneva score.^32^ Jian Liu et al. found that the Wells score had a sensitivity of 88.72 % and a specificity of 35.37 % for diagnosing APE.^33^ The results of the present study are broadly similar to these previously reported findings, which shows that this classic score still retains high clinical application value.

There are some limitations to this study. The differences in aggravating duration of COPD and smoking time may affect the results of coagulation parameters. Second, the study cohort consisted of a relatively small number of subjects and relied on self-reported information from the subjects, which may limit the generalizability of the findings. The study utilized a case-control study, which can only establish association, not causation. Future studies should aim to include more diverse and representative samples, and adding larger sample sizes could provide more reliable results and better overall representation.

In summary, this study emphasized a significant association between the occurrence of PE in patients with AECOPD and the thrombin generation indices ETP, ttpeak and ATPP as well as the Wells score. Based on this study, it can be used as a diagnostic strategy for PE in patients with AECOPD.

## Data availability

The data that support the findings of this study are available from the corresponding author, upon reasonable request.

## Ethics statement

The present study was approved by the Ethics Committee of Wenjiang District People's Hospital of Chengdu (IRB20230428) and written informed consent was provided by all patients prior to the study start. All procedures were performed in accordance with the ethical standards of the Institutional Review Board and The Declaration of Helsinki, and its later amendments or comparable ethical standards.

## Authors’ contributions

Conceptualization, Linjie Luo and Dan Zheng; methodology, Li Da and Jian Cheng; formal analysis, Yirui Cao and Na Wang; investigation, Li Da and Yirui Cao; data curation, Jian Cheng and Na Wang; writing-original draft preparation, Linjie Luo and Dan Zheng; writing-review and editing, Linjie Luo. All authors have read and agreed to the published version of the manuscript.

## Funding

Sichuan Medical Association Youth Innovation Project (n° Q21048).

## Conflicts of interest

The authors declare no conflicts of interest.
